# Eave and swarm collections prove effective for biased captures of male *Anopheles gambiae* mosquitoes in Uganda

**DOI:** 10.1186/s13071-021-04770-x

**Published:** 2021-05-26

**Authors:** Krystal Birungi, Danspaid P. Mabuka, Victor Balyesima, Annet Namukwaya, Elinor W. Chemoges, Sylvia Kiwuwa-Muyingo, C. Matilda Collins, Frederic Tripet, Jonathan K. Kayondo

**Affiliations:** 1 Entomology Division, Uganda Virus Research Institute (UVRI), Plot 51-59, P.O. Box 49, Entebbe, Uganda; 2 MRC/UVRI & LSHTM Uganda Research Unit, Plot 51-59, P.O. Box 49, Entebbe, Uganda; 3 The Centre for Environmental Policy, Imperial College London, The Weeks Building, 16-18 Princes Gardens, London, SW7 1NE UK; 4 Centre for Applied Entomology and Parasitology, School of Life Sciences, Keele University, Staffordshire, ST5 5BG UK

**Keywords:** Malaria, Mosquito sampling, Vector ecology, Resting traps, Swarm sampling, Aspiration, Eave

## Abstract

**Background:**

Traditional malaria vector sampling techniques bias collections towards female mosquitoes. Comprehensive understanding of vector dynamics requires balanced vector sampling of both males and females. Male mosquito sampling is also necessary for population size estimations by male-based mark-release-recapture (MRR) studies and for developing innovations in mosquito control, such as the male-targeted sterile insect technique and other genetic modification approaches. This study evaluated a range of collection methods which show promise in providing a more equal, or even male-biased, sex representation in the sample.

**Results:**

Swarms were found at all study sites and were more abundant and larger at the peak of the wet season. Swarm sampling caught the most males, but when man/hour effort was factored in, sampling of eaves by aspiration was the more efficient method and also provided a representative sample of females. Grass-roofed houses were the most productive for eave collections. Overall few mosquitoes were caught with artificial resting traps (clay pots and buckets), although these sampling methods performed better at the start of the wet season than at its peak, possibly because of changes in mosquito ecology and an increased availability of natural resting sites later in the season. Aspiration of bushes was more productive at the peak of the wet season than at the start.

**Conclusions:**

The results of this study demonstrate that eave aspiration was an efficient and useful male mosquito collection method at the study sites and a potentially powerful aid for swarm location and MRR studies. The methods evaluated may together deliver more sex-balanced mosquito captures and can be used in various combinations depending on the aims and ecological parameters of a given study.

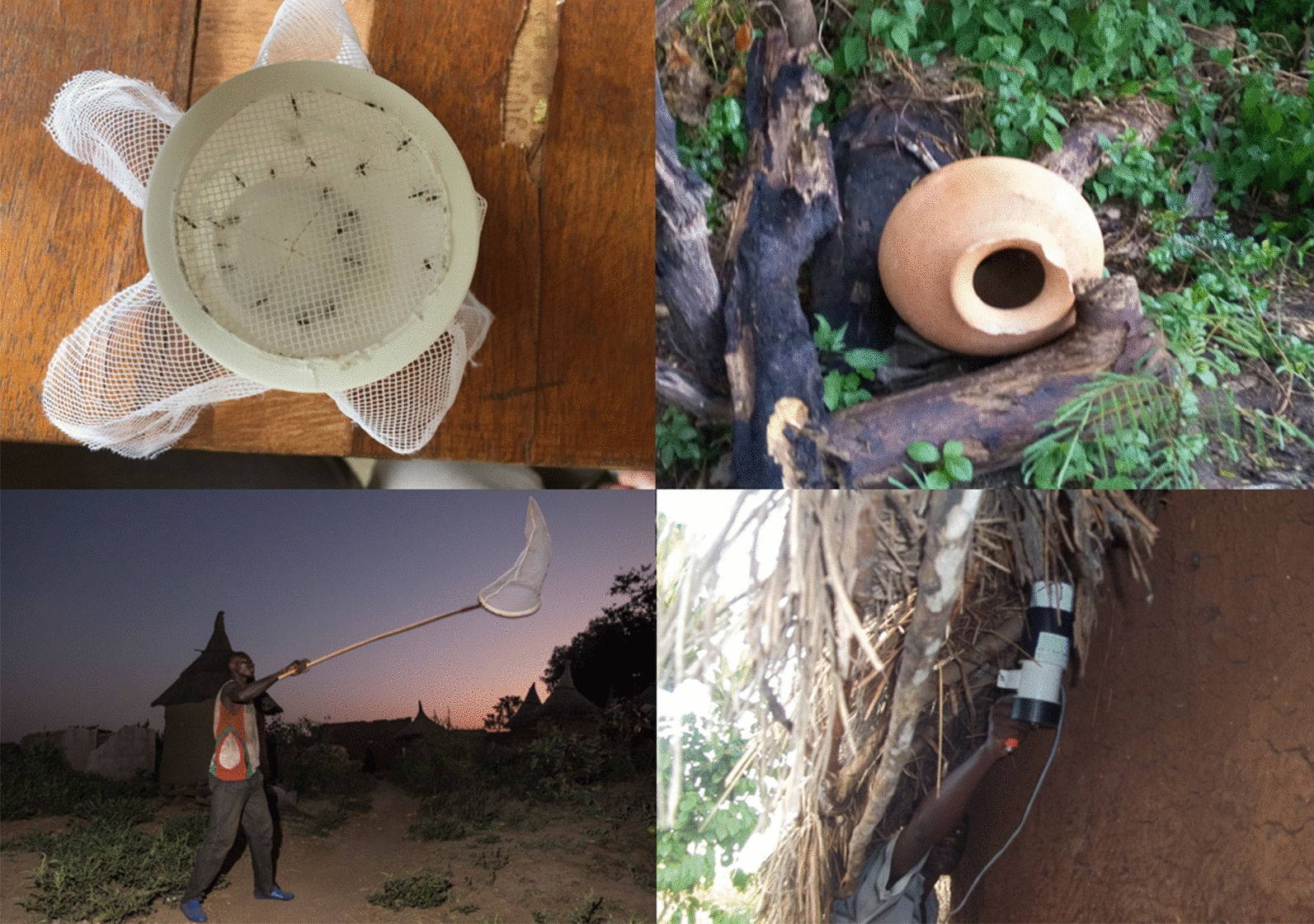

## Background

Malaria prevention requires studying vector species to understand disease transmission and to effectively implement appropriate vector control. Although malaria control efforts have intensified over the past 10 years, with millions of lives saved [1, 2], the effectiveness of mainstay methods, such as insecticide-treated nets (ITNs) and indoor residual spraying (IRS), is decreasing [3, 4]. Insecticide resistance is increasing in mosquito populations, and new tools are urgently needed [5–9]. With new technologies being proposed, such as active genetics (gene drives), and other genetic modification products, it is important to have an understanding of the ecology and biology of vector species that is supported by consistent species-wide sampling techniques [7, 8, 10]. Consistency in these methods will enable the development of comparable baselines and the evaluation of novel interventions.

Entomological surveillance and population studies for malaria control require comprehensive and strategized field collections for sampling mosquito vectors [11]. To effectively sample mosquito populations, different collection methods and equipment are deployed depending on the aim of the study and the type of information/data required. A variety of collection methods are currently available for sampling adult and immature mosquito stages, which have been described in literature [12, 13]. Ideally, a representative sample of an adult vector population would contain unfed, blood-fed and gravid females as well as males. However, although a large body of work on mosquito surveillance has been compiled, emanating from decades of vector research and control efforts, the majority of the trapping techniques currently in use are inherently and explicitly targeted at the capture of females—most likely due to the fact that malaria vector control has also focused on the plasmodium-spreading female mosquitoes instead of the males which do not bite.

Mosquito sampling for surveillance and research studies must provide reliable estimations of parameters such as distribution, density and abundance of both indoor and outdoor populations [14]. Indoor resting *Anopheles gambiae* are commonly sampled using knockdown spray catches, which involves the spraying of insecticide into a room to knock down individuals onto a sheet, enabling collection by hand, or by using mechanical aspirators [15, 16]. These approaches predominantly recover females resting after a blood meal and only a small proportion of males. Many collection methods, including those used for outdoor sampling, rely on lures that can be generally classified into biological (human/animal baited collections), chemical (various molecular baits, such as CO_2_), physical (light, heat or color) and combined physicochemical attractants [9, 17, 18]. These also attract mostly host-seeking females in flight that are lured by visual, olfactory and thermal cues, or gravid females seeking to oviposit.

Female *Anopheles* mosquitoes transmit the malaria parasite when taking a blood feed, males feed only on sugar sources, such as nectar from plants [19]. Thus, mosquito collections conducted with the aim of understanding malaria transmission have traditionally been carried out with tools designed to maximize female captures; however, the collection of male mosquitoes is becoming necessary for many scenarios. For example, when estimating population size by mark-release-recapture (MRR) studies, from an ethical perspective it may be considered more acceptable to make use of males rather than females. Females are a biting nuisance and are potential malaria vectors; also they will probably contribute offspring to the next generation. With collection methods so skewed towards female mosquito collections, it is a possibility that the lack of sufficient males in the catch may skew the arising population size estimates. The sterile insect technique (SIT) and novel male-mediated genetic control approaches have been proposed for *Anopheles* mosquitoes [20]. Studies to better understand male ecology and reproductive biology in many ecological regions are essential for optimization and future field evaluation of the efficacy of such techniques.

In this study we evaluated a range of mosquito collection methods which might mitigate the female sampling bias. Published literature on and reviews of previous mosquito capture studies were explored and those with the potential for male capture were identified. Other than swarm captures, which are inherently male-targeted, methods focusing on mosquito resting shelters, such as vegetation and eaves, were considered to be potentially productive places to find males, but required empirical assessment [21]. Therefore, in addition to swarm collections, the study focused on clay pot traps, resting bucket traps, house eaves and bush aspiration, all methods which have shown varying degrees of promise for male mosquito capture in other settings [22, 23]. Collections were performed in three mainland villages in Uganda and the methods of capture compared in terms of the species captured, the sex ratio of *A. gambiae* samples and females and males capture rates per unit of capture and/or time.

## Methods

### Study design

In Uganda, the prevalent mosquito species responsible for the spread of malaria is *Anopheles gambiae* (*s.l.*) (*A. gambiae*) [24]. Five mosquito collection methods, all previously identified for their potential to catch male *A. gambiae* according to the literature review, were selected for evaluation at three village study sites in mainland Uganda. In 2017, two rounds of collections were made. The first collection round was at the start of the rainy season, targeting the early mosquito population period, and the second was at the peak of the rainy season, targeting higher mosquito numbers.

### Study sites and descriptions

**Fig. 1:**
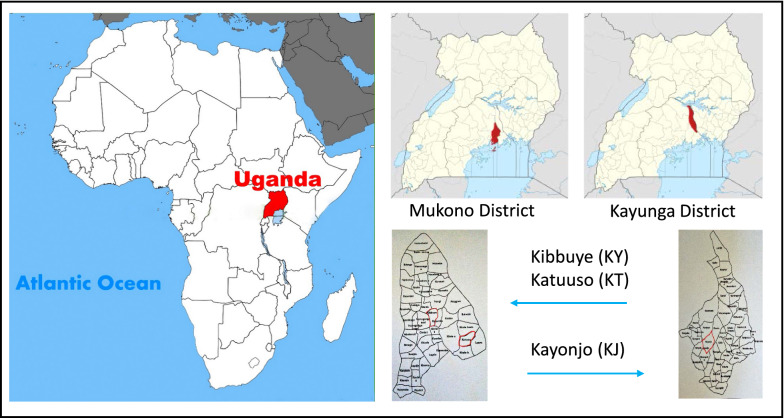
Location of the study sites in Uganda. The study villages of Kibbuye (*KY*) and Katuuso (*KT*) are in Mukono district and Kayonjo (*KJ*) is in Kayunga district

The study villages were Kibbuye (KY) and Katuuso (KT) in Mukono district, and Kayonjo (KJ) in Kayunga district (Fig. 1). The villages are located in central Uganda and typically experience two rainy seasons and two dry seasons per year. The first rainy season is generally from March to June, followed by a dry season from July to September. The second shorter rainy season runs from October to November and is followed by a dry period from December to February. All three sites record high malaria incidence (up to 150 confirmed malaria cases per 1000 population/year) and are located in areas in Uganda that have high malaria endemicity [24].

#### Kibbuye village

Kibbuye village is located in Seeta-namuganga subcounty, Mukono district (0.724°N, 32.784°E) and has approximately 1500 inhabitants. The major economic activity is agriculture, and residents plant rice gardens in the swampy areas bordering the village and coffee plants within the village. In addition to crop farming, some villagers also keep livestock such as cows, pigs and goats on a small scale, with each family owning on average fewer than ten animals. The major bushy vegetation is coffee plants, which are abundant throughout the village. Most mosquito larval habitats are found in the rice gardens but they are also found in local rock pools of collected rainwater.

#### Katuuso village

Katuuso village is also located in Seeta-namuganga subcounty, about 7 km southeast of Kibbuye (0.699°N, 32.843°E), Katuuso has approximately 800 inhabitants. The major economic activity is again agriculture, with gardens located throughout the swampy areas bordering the village. While there is some rice farming in Katuuso village, various annual food crops, such as sweet potatoes and maize, are also common. A few families also keep cows, pigs and goats, owning on average, fewer than five animals. As in Kibbuye, coffee plants are abundant in Katuuso, although on a smaller scale. The mosquito larval habits are located in the crop gardens.

#### Kayonjo village

Kayonjo village is located in Busaana subcounty of Kayunga district (0.925°N, 32.862°E) and has approximately 1800 inhabitants. Kayonjo is located approximately 10 km east of Kibbuye and Katuuso. It is similar to the other study villages in that the major economic activity is agriculture and the areas bordering the village are mostly large tracts of swampy ground on which residents farm. Agriculture here is diverse, with a wide range of food crops such as maize, sweet potatoes, rice and yams. Coffee plants are also abundant in addition to large evergreen trees. Most trees are fruit trees with mango trees especially plentiful. Livestock farming in Kayonjo is mainly on a subsistence basis with most households owning only one or two cows. The swampy gardens once again contain the majority of mosquito larval habitats.

### Sampling approach

Two field sampling visits were made in the three villages, the first at the start of the rainy season and the second at its peak. The trips took place from 27 March to 8 April 2017 (start of rainy season) and from 22 May to 3 June 2017 (peak of the rainy season).

The study sites were sampled sequentially during each collection trip, starting with Kibbuye village and ending with Kayonjo village. Each village was sampled over a 2-day period during each collection trip using the methods described below. The locations sampled were the same for each visit. Swarm sampling took place at dusk, but for all other methods, sampling took place in the early morning, which is considered optimal for mosquito collection [25]. For some methods (EAV, CPT, RBT and BUSH, see below), repeat collections at the same sites took place later in the day, typically early to mid-afternoon in in an effort to ascertain whether mosquitoes continued to move to resting places later in the morning and could have been missed in the initial early morning collection.

#### Aspiration of eaves

**Fig. 2:**
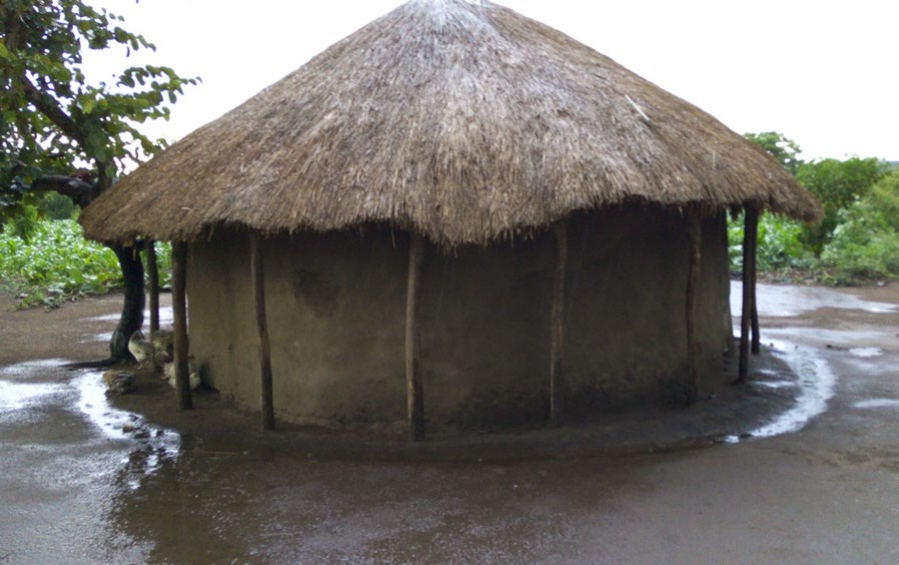
Typical house used for aspiration of eaves. Of the 30 houses selected for eave aspiration, 24 had mud walls with grass-thatched, overhanging roofs and six were similar but with iron-sheet roofing material

Ten houses, evenly distributed around each village, were selected for mosquito collection using the aspiration of eaves (EAV) method. The village was stratified into five approximately equal sections, then two grass-thatched houses (Fig. 2) were randomly selected from each section. In cases where no grass-thatched house was available, iron sheet-roofed houses with mud walls were selected. Each house was sampled early in the morning (06:00 h) which is the optimal time to capture resting mosquitoes. Two collectors equipped with Prokopack aspirators (model 1419; John W. Hock Co., Gainesville, FL, USA) collected mosquito samples in each village, one collector per house. A timed 10-min sampling system was used to ensure standardization of the collection effort. The collector tried to aspirate the entire eave area as exhaustively as possible by moving the aspirator nozzle back and forth over the collection area while slowly moving around the house. Samples were then labeled and stored for later identification and analysis.

#### Indoor aspiration of houses

In each village ten houses were selected for mosquito collection using the indoor aspiration of houses (ASP) method by stratified randomization to proportionately represent the house types and spatial area of the village (Table 1). Three house types were present in Katuuso and Kibbuye (Table 1). In Kayonjo, fired bricks are not used as construction material for domestic buildings but reserved for community buildings such as schools and religious buildings. Aspiration started at 06:00 h. If houses had more than one interior room, only one room (the sleeping room) was aspirated. Two collectors equipped with Prokopack aspirators collected mosquito samples in each village. A timed 10-min sampling system was used. The collector aspirated the entire room as exhaustively as possible by moving the aspirator back and forth over the walls, interior eaves, under furniture and over the interior of the roof. Samples were then labeled for later identification and analysis.

**Table 1:** Distribution of house types where indoor (interior) and eave aspirations were performed in the three villages

Village	House type					
	Walls: mud brick		Walls: mud brick		Walls: brick	
	Roof: grass thatch		Roof: iron sheets		Roof: iron sheets	
	Interior	Eaves	Interior	Eaves	Interior	Eaves
Kibbuye	2	7	4	3	4	0
Katuuso	1	8	6	2	3	0
Kayonjo	2	9	8	1	0	0
All	5	24	18	6	7	0

#### Aspiration of clay pots and resting bucket traps

**Fig. 3:**
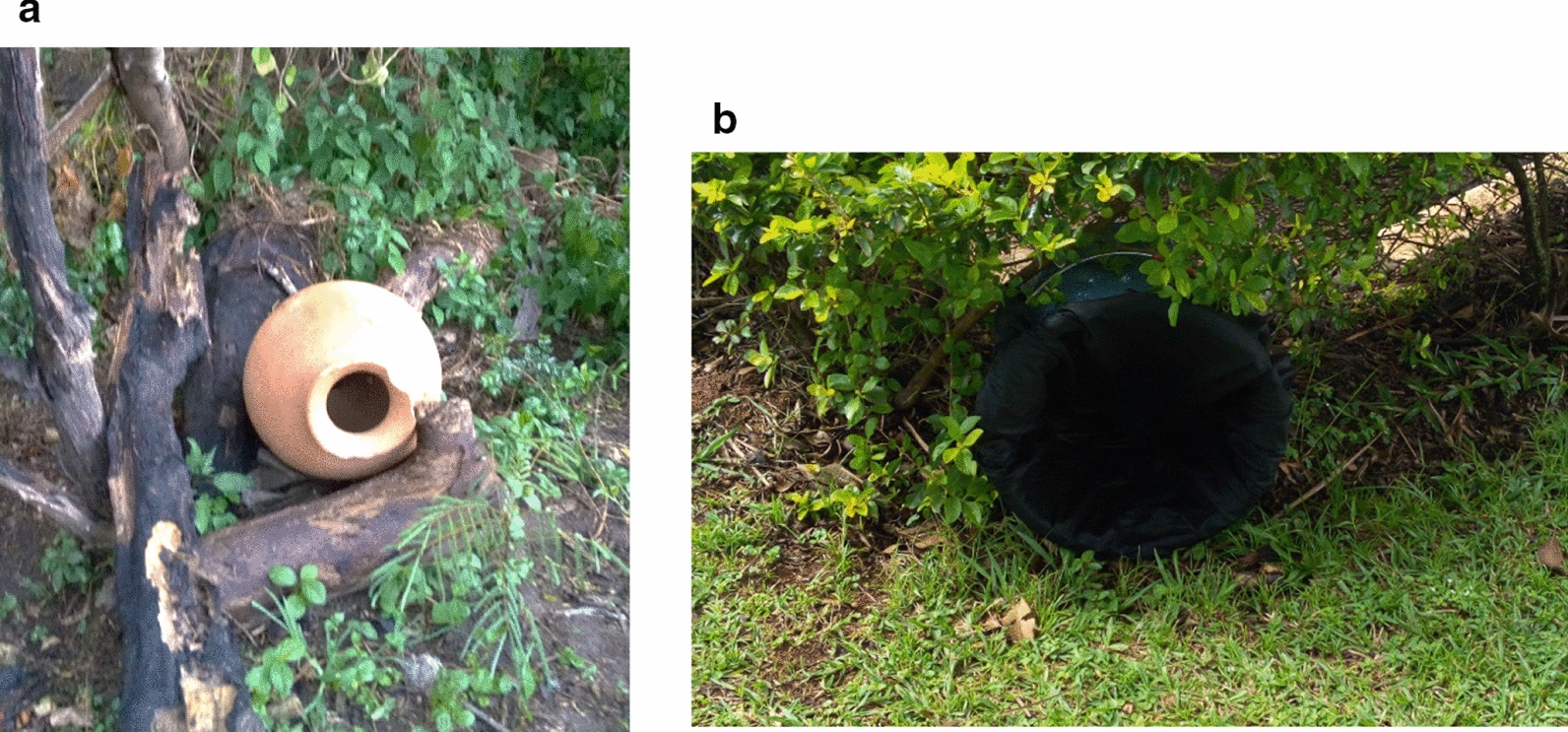
Clay pot (**a**) and resting bucket trap (**b**). These were deployed in pairs, 5 m from each other, on opposing sides of selected houses throughout each village

For the aspiration of clay pots (CPT) method, ten clay pots were locally manufactured following the design adopted from Odiere et al. [22]. This design was slightly modified by reducing the pot mouth to 10 cm in diameter from 20 cm (Fig. 3). The pots were unpainted, fired clay of approximately 20-l capacity. Holes (diameter 2 cm) were bored into the bottom of the pots to discourage theft by making the pots unable to store water.

Ten resting bucket traps (RBTs) (Fig. 3) were made following guidelines of Kreppel et al. [26]. A standard 20-l plastic bucket was lined with wet, black cotton fabric to increase humidity. The buckets were modified by puncturing nail holes in the bottom and sides to discourage theft as described above.

Clay pots and RBTs were placed 5 m from each house used for indoor aspiration (Table 1) but on opposite sides and left overnight before the first sample was taken. One collector equipped with a Prokopack aspirator collected mosquitoes from each trap type in each village. A timed, 2-min sample was taken from each pot. The aspiration started at the mouth of the pot, gradually moving deeper within the pot. Aspiration of the resting shelters was carried out between 06:00 h and 08:00 h. Samples were then labeled for later identification and analysis.

#### Aspiration of bushes

The aspiration of bushes (BUSH) method was used to collect mosquitoes from 15 bushes around each village using a CDC backpack aspirator (model 1412; John W. Hock Co.). Bushes were selected in a stratified random way by location to provide even coverage of the village area. Bushes were mainly midsized shrubs approximately 1 m in height (or less) and 1 m across (or less). Bush aspirations were carried out between 06:00 h and 08:00 h by a single collector in each village. The collector took a 15-min aspiration sample from each bush moving from top to bottom while slowly circling the plant. Samples were then labeled for later identification and analysis.

#### Swarm collection sampling

For swarm collection sampling (SWN), each village was notionally divided into two halves and mosquitoes were collected from each half during two sequential days in each survey. Swarm collections were made following the method described by Diabate et al. [27]. Swarm collectors worked in pairs, locating points of contrast on the ground at around 18:00 h and watching the spaces above these markers against the lighter background of the sky at dusk. When a swarm was seen, the collectors made multiple sweeps of the swarm using a sweep net until the end of the swarming period at complete sunset. Each net with a collection was labeled with the location of the swarm, estimated swarm size, height of swarm above ground and any landmark associated with it, and then stored for later identification. The following morning, the collected mosquitoes were aspirated and, if still alive, killed with chloroform.

### Mosquito identification and processing

All collected mosquitoes were identified to morpho-species in the field by a trained entomologist equipped with a microscope and a morphological key [28]. Identified samples were then placed in a clearly labeled 1.5-ml tube and stored in 80% ethanol for transport to the laboratory at the Uganda Virus Research Institute (UVRI) for molecular confirmation of morphological identification by PCR using the protocol described by Wilkins et al. [29]*.* The PCR cycling conditions were melting at 95 °C for 5 min; then 95 °C/30 s, 58 °C/30 s 72 °C/30 s for 30 cycles; followed by 1 cycle of 72 °C for 5 min. Each reaction comprised template DNA (2 ng), primers (1 µM), MgCl_2_ (0.3 mM), dNTPs (0.08 mM), Taq polymerase (1U), Go Green Taq buffer (1×) and distilled H_2_0 topped to 25-μl total reaction volume. PCR products (10 ul) were observed by separation on agarose (1%) TBE gels run in 0.5× TBE buffer at 12 V/cm, and fragment sizes were estimated using a 1-kb ladder marker. The primers used were IMP-UN, QD-3T, ME-3T GA-3T and AR-3T.

### Statistical analysis

All collected data were analyzed using JMP version 14 software (SAS Institute, Inc., Cary, NC, USA) [30]. Analyses were conducted using parametric and non-parametric methods as appropriate.

For each method of capture and season, the sum of captured *A. gambiae* (*s.l.*) male and female mosquitoes, as well as those of *Aedes* spp., *Culex* spp. and *Mansonia* spp., was calculated. Some differences in mosquito abundance were expected among the villages sampled arising from, for example, their geography and water availability, but their extent could not be predicted. In order to estimate the influence of any variation detected, appropriate linear models with main effects and interaction terms were fitted to the data. Non-significant interactions were removed from models following a stepwise approach.

#### Total number of *A. gambiae* (*s.l.*) captured

The total number of individuals captured per method were compared using frequency tests and likelihood test on odds ratios (ORs) for the data collected during early and peak rainy seasons.

#### Proportion of males

Pairwise post-hoc comparisons between methods were performed using likelihood tests on ORs. In order to identify which methods produced sex-biased or more balanced catches, as distinct from the numbers caught, the proportion of *A. gambiae* males captured was investigated as a function of village, season and method of capture using a binomial generalized linear model (GLM). The data was also analyzed for each season separately. Following this, two dependent variables particularly relevant to field entomologists were calculated and analyzed. The first was the number of females and males captured per unit of collection method, i.e. per room, eaves, per bush aspirated or per trap (clay pot, resting bucket) or per swarm netted. This variable therefore informs the number of collections needed with a given method to collect a given number female or male mosquitoes (e.g. number of house aspirations or clay pots). The second variable takes into account the human resources needed by estimating the yield or number of mosquitoes per unit of collection described above, calculated per number of man hours required to perform such sampling.

#### Mosquito numbers caught per collection

The influence of the factors village, method and season, and their interaction, on the number of females and males caught per unit of collection was analyzed using GLMs with Poisson distribution and correction for over-dispersion. The data was also analyzed for each season separately. The statistical significance of interactions was tested, but these interactions were removed in a stepwise manner if non-significant. Post-hoc pairwise group comparisons were performed using model contrasts.

#### Yield (mosquitoes caught per man hour)

Yield was analyzed using GLMs with Poisson distribution and correction for over-dispersion. The data were also analyzed for each season separately. The statistical significance of interactions was tested but these interactions were removed in a stepwise manner if non-significant. Post-hoc pairwise group comparisons were performed using model contrasts.

#### Analysis of mosquito yield by house type

Because of greatly imbalanced sample sizes, unequal variance and non-normality, non-parametric Kruskal–Wallis tests were used to test the effect of each house type on the female and male yield per hour for indoor indoor and eave aspirations.

## Results

### Afternoon sampling

In one of 223 repeat samples taken in the early afternoon, only one mosquito was found (1 female *A. gambiae*), collected using the EAV method. These afternoon samples, which strongly indicate a lack of daytime mobility in these mosquitoes, were therefore not included in the general data frame.

### Overall catch counts and species composition

**Fig. 4:**
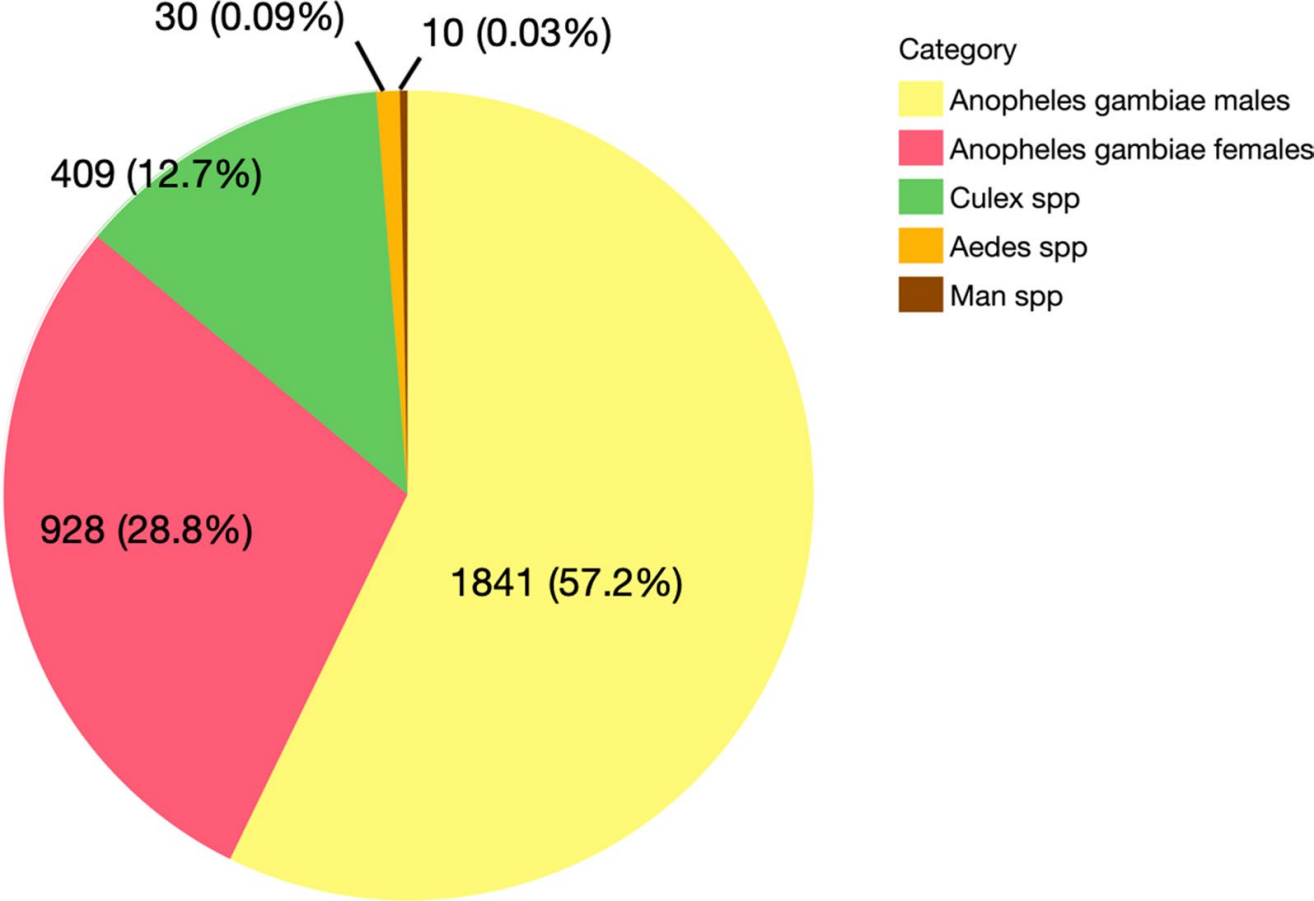
Number and percentage of female and male *Anopheles gambiae* (*s.l.*) mosquitoes and other mosquito species captured during the entire study period.* Man* *Mansonia*

A total of 2769 mosquitoes were caught during the two surveys, of which 86% (*n* = 2769) were *A. gambiae* (*s.l.*), 13% (*n* = 409) were *Culex* spp., < 1% (*n* = 30) were *Aedes* spp. and < 1% (*n* = 10) were *Mansonia* spp. (Table 2; Fig. 4)*.* Of the *A. gambiae* (*s.l.*) mosquitoes captured, 67% were male (*n* = 1841) and 33% were female (*n* = 928) (Table 2), reflecting that our choice of methods was geared towards male captures. The mosquitoes captured from Katuuso and Kibbuye villages comprised 100% *A*. *gambiae*; in contrast, only a very small percentage of the mosquitoes captured in Kayonjo village were *A*. *arabiensis* (< 1%, *n* = 12), with the rest made up of *A. gambiae.*

**Table 2:** Mosquito samples collected throughout the study

Season	Village	Method^a^	*N*		Sum (*Anopheles gambiae*, both sexes)		Sum (*A. gambiae*, females)		Sum (*A. gambiae*, males)		Sum (*Culex*.spp.)		Sum (*Mansonia* spp.)		Sum (*Aedes* spp)	
Start of rainy season	KJ	ASP	10		128		110		18		8		1		0	
		BUSH	15		0		0		0		1		0		0	
		CPT	10		0		0		0		0		0		0	
		EAV	10		115		46		69		4		1		1	
		RBT	10		0		0		0		0		0		0	
		SWN	8		128		0		128		0		0		0	
	KT	ASP	9		24		24		0		2		0		0	
		BUSH	15		3		1		2		3		0		0	
		CPT	10		1		1		0		0		0		0	
		EAV	10		43		22		21		1		1		1	
		RBT	10		4		3		1		0		0		0	
		SWN	15		214		0		214		11		0		0	
	KY	ASP	11		60		50		10		6		0		0	
		BUSH	15		5		2		3		34		3		4	
		CPT	10		1		0		1		0		1		0	
		EAV	10		14		8		6		0		0		0	
		RBT	10		0		0		0		2		0		0	
		SWN	12		111		4		107		81		1		23	
Start of rainy season—total			200		851		271		580		153		8		29	
Peak of rainy season	KJ	ASP	10		155		138		17		21		1		1	
		BUSH	15		23		4		19		1		0		0	
		CPT	10		9		2		7		0		0		0	
		EAV	10		380		92		288		19		0		0	
		RBT	10		4		3		1		0		0		0	
		SWN	8		109		0		109		0		0		0	
	KT	ASP	10		198		174		24		24		1		0	
		BUSH	15		9		1		8		19		0		0	
		CPT	10		5		2		3		1		0		0	
		EAV	10		80		28		52		7		0		0	
		RBT	10		5		0		5		17		0		0	
		SWN	15		214		0		214		11		0		0	
	KY	ASP	11		115		99		16		65		0		0	
		BUSH	15		14		9		5		15		0		0	
		CPT	10		1		0		1		0		0		0	
		EAV	10		255		104		151		43		0		0	
		RBT	10		8		1		7		6		0		0	
		SWN	12		334		0		334		7		0		0	
Peak of rainy season—total			201		1918		657		1261		256		2		1	

*KJ* Kayonjo village, *KT* Katuuso village, *KY* Kibbuye village

^a^*ASP* Indoor aspiration of houses,* BUSH* aspiration of bushes,* CPT* aspiration of clay pots,* EAV* aspiration of eaves,* RBT* resting bucket traps,* SWN* swarm collection sampling

### Effect of method on number and male proportion of *A. gambiae* (*s.l.*) captured

**Fig. 5:**
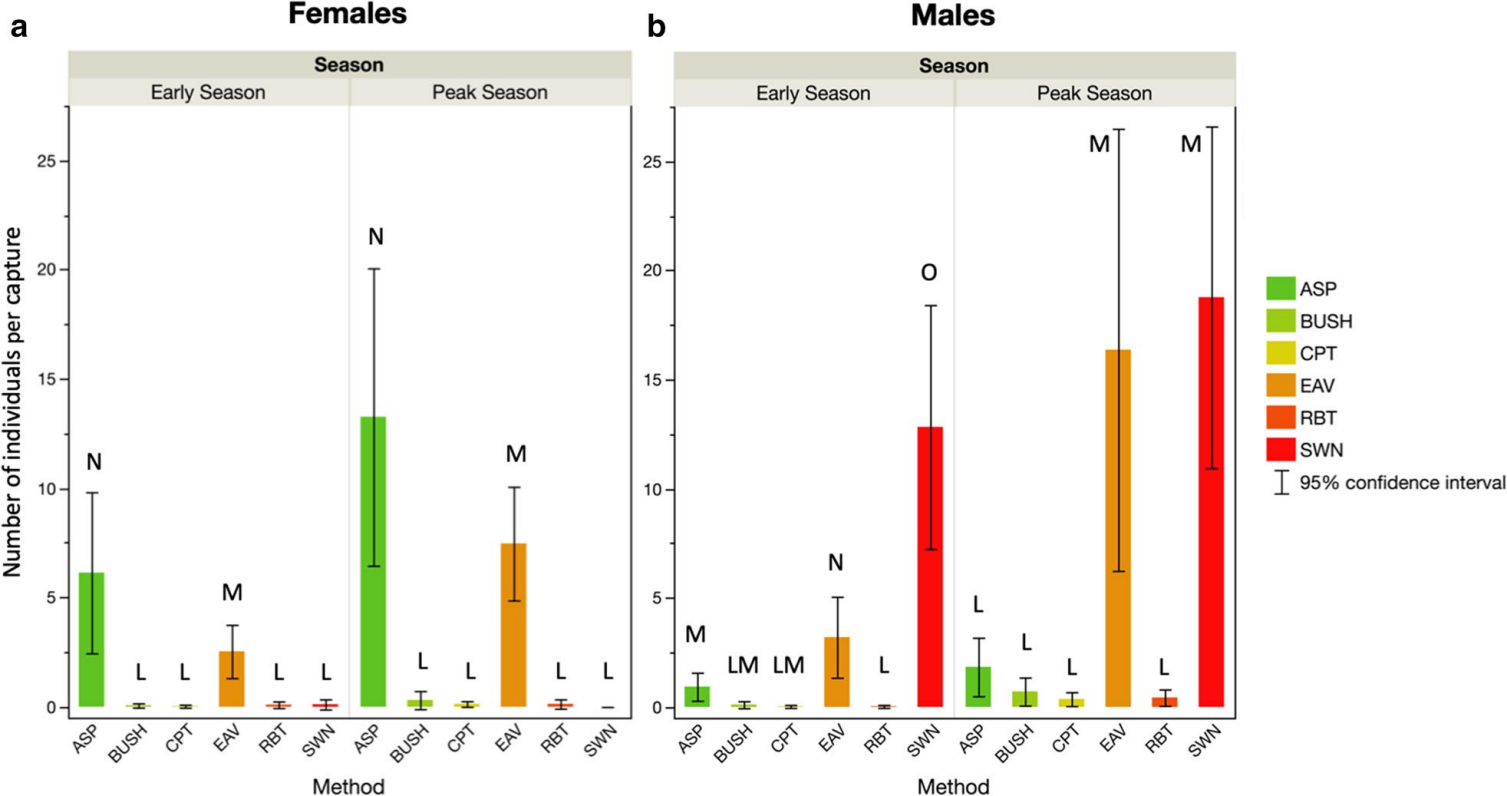
The mean number of females (**a**) and males (**b**) per house, eave clay pot, bush, bucket or swarm by season. See footnote of Table 2 and section “Sampling approach” for definition of abbreviations description of the sampling methods used. Bars labelled with different letters were significantly different (model contrast: P < 0.05)

A total of 851 *A. gambiae* (*s.l.*) were captured at the start of the rainy season (Table 2; counts per village, per season per method). Overall, a significantly larger proportion of individuals (53%) were collected using SWN than with the other methods (likelihood test on ORs: *P* < 0.0001). The EAV and ASP methods caught 25 and 20% of the total number of *A. gambiae* (*s.l.*), respectively; these two methods differed significantly in terms of number captured from each other and from the other methods (*P* < 0.001 in all comparisons). The least effective methods of capture were the BUSH (1%), RBTs and CPTs methods (both < 1%); these latter two methods did not differ in terms of number of *A. gambiae* (*s.l.*) caught (*P* > 0.05) (Table 2; Fig. 5). A much larger number of *A. gambiae* (*s.l.*) (*n* = 1918) were captured at the peak of the rainy season (Table 2). At that time, the EAV and SWN methods captured comparable numbers of individuals (37 and 34%, respectively) (*P* = 0.1175), with ASP the next most effective method (25%). The least effective methods of capture were again the BUSH (2%) and RBT and CPT methods (both < 1%) (Table 2; Fig. 5).

Overall, the proportion of males caught did not vary between the three villages but it did change with the season, increasing from 66% in the early season to 68% at the peak season (*P* < 0.001) (Tables 3, 4). The different methods of capture resulted in different proportions of males although these proportions varied slightly between villages (*P* < 0.001) (Tables 3, 4). A comparison of all capture methods showed that overall, a lower proportion of males was caught with the ASP method (likelihood test on ORs: *P* < 0.0001 in all pairwise comparisons) and a greater proportion of males was caught with the SWN method (*P* < 0.0001 in all cases) (Fig. 5). The proportion of males captured by all other sampling methods was intermediate and there was no evidence of variation among them (*P* > 0.3511 in all cases) (Fig. 5). Separate independent analyses of the early and peak seasons led to the same conclusions on these methods of capture.

**Table 3:** Summary statistics of the binomial generalized linear model used to estimate the influence of season, village and method on the proportion of *Anopheles gambiae* male mosquitoes captured

Source	*df*	L–R Chi square^a^	*P*-value
Season	1	13.5	0.0002
Village	2	0	1.000
Method	5	1657.2	< 0.0001
Village × Method	8	4.5	0.0003

Main effects and statistically significant, or near-significant, interactions are shown

^a^Likelihood ratio Chi-squared statistic

**Table 4:** Post-hoc pairwise comparisons (likelihood test on odds ratios) of the proportion of *A. gambiae* female and male mosquitoes collected by the different methods

Comparison	Odds ratio	*P*-value	Lower 95% CI	Upper 95% CI
BUSH* vs* ASP	16.24	< 0.0001	7.95	33.19
CPT* vs* ASP	10.77	< 0.0001	4.09	28.35
CPT* vs* Bush	0.66	0.4876	0.21	2.11
EAV* vs* ASP	11.66	< 0.0001	8.82	15.43
EAV* vs* Bush	0.72	0.3511	0.36	1.44
EAV* vs* CPT	1.08	0.8704	0.42	2.81
RBT* vs* ASP	12.55	< 0.0001	4.53	34.78
RBT* vs* BUSH	0.77	0.6746	0.23	2.57
RBT* vs *CPT	1.17	0.8266	0.30	4.57
RBT* vs *EAV	1.08	0.8863	0.39	2.94
SWN* vs* ASP	996.49	< 0.0001	466.43	2128.92
SWN* vs* BUSH	61.37	< 0.0001	22.77	165.37
SWN* vs* CPT	92.49	< 0.0001	28.30	302.24
SWN* vs *EAV	85.43	< 0.0001	40.71	179.28
SWN* vs* RBT	79.39	< 0.0001	23.26	270.96

*CI* Confidence interval

### Number of *A. gambiae* (*s.l.*) mosquitoes caught per collection method

**Fig. 6:**
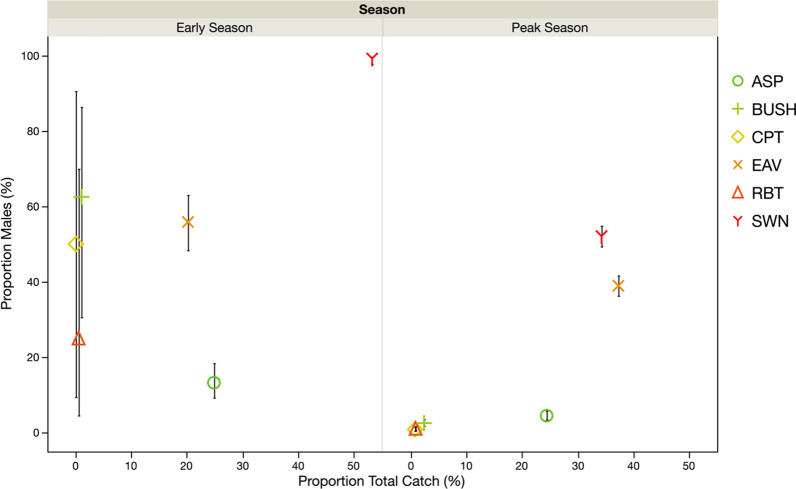
Proportions of *A. gambiae* (*s.l.*) caught using the different methods and corresponding proportion (%) of male mosquitoes captured, by season. The error bars indicate the confidence intervals around the estimates of sex ratio

Overall, the numbers of male mosquitoes caught per unit (sample) of each collection method (e.g. per house aspiration, per clay pot, per swarm) varied between villages, but there was no evidence of this variation for female mosquitoes (Tables 5, 6). The numbers of *A. gambiae* male and female mosquitoes captured per method increased from the early to peak rainy season. There was evidence of variation in the response to season between villages for the numbers of males, but not for females (Tables 5, 6). For both sexes, the method used explained by far the largest proportion of variation in the numbers collected per unit (Tables 5, 6; Fig. 6). For females, more individuals were captured with the ASP method than with any of the other methods, and this was true in both seasons (model contrasts: *χ*^2^ > 75.0 *P* < 0.0002 in all pairwise comparisons). The next most productive method was the EAV method, which caught higher numbers than the remaining methods in both seasons (*χ*^2^ > 17.1, *P* < 0.0001 for all significant comparisons) (Fig. 6). There was no evidence of variation between the RBT and CPT methods, swarm collections (SWN) and the BUSH method, which all captured low numbers of female mosquitoes regardless of season (model contrasts: *P* > 0.05 in all comparisons) (Fig. 6).

**Table 5:** Summary statistics of the generalized linear model (Poisson distribution with over-dispersion) used to estimate the influence of season, village and method on the number of *A. gambiae* female and male mosquitoes collected per unit of collection (room, eaves, bush, bucket, pot, swarm)

Sex	Source	*df*	L–R Chi square^a^	*P*-value
Females	Season	1	23.3	< 0.0001
	Village	2	5.7	0.0593
	Method	5	288.1	< 0.0001
Males	Season	1	7.6	0.0058
	Village	2	8.4	0.0152
	Method	5	281.7	< 0.0001
	Season × Village	2	7.7	0.0215
	Season × Method	5	12.2	0.0325

Main effects and statistically significant, or near-significant, interactions are shown

^a^Likelihood ratio Chi-squared statistic

**Table 6:** Post-hoc pairwise comparisons (generalized linear model contrasts) of the number of *A. gambiae* female and male mosquitoes collected per unit of collection (room, eaves, bush, bucket, pot, swarm) using different methods

Comparison	Value	Log-likelihood	Chi-square	*P-*value
Females				
BUSH–ASP	3.94	200.41	137.62	< 0.0001*
CPT–ASP	4.76	187.49	111.78	< 0.0001*
CPT–BUSH	0.82	131.81	0.43	< 0.0001*
EAV–ASP	0.67	138.44	13.68	= 0.0002*
EAV–BUSH	3.28	163.47	63.75	< 0.0001*
EAV–CPT	− 4.09	158.87	54.54	< 0.0001*
RBT–ASP	4.42	186.34	109.49	< 0.0001*
RBT–BUSH	0.48	131.69	0.18	= 0.6721
RBT–CPT	− 0.34	131.62	0.05	= 0.8246
RBT–EAV	3.76	157.91	52.63	< 0.0001*
SWN–ASP	5.09	192.27	121.34	= 0.4325
SWN–BUSH	1.15	131.98	0.77	= 0.3805
SWN–CPT	0.33	131.62	0.04	= 0.8501
SWN–EAV	4.42	161.62	60.06	< 0.0001*
Males				
BUSH–ASP	1.54	–^a^	3.32	= 0.0685
CPT–ASP	2.48	–	2.13	= 0.1448
CPT–BUSH	0.93	–	0.26	= 0.6092
EAV–ASP	− 1.71	–	17.57	< 0.0001*
EAV–BUSH	− 3.25	–	17.18	< 0.0001*
EAV–CPT	− 4.18	–	6.29	= 0.0122
RBT–ASP	2.39	–	2.01	= 0.1563
RBT–BUSH	0.85	166.04	0.29	= 0.5937
RBT–CPT	− 0.08	165.90	0.001	= 0.9715
RBT–EAV	4.10	–	6.12	= 0.0134
SWN–ASP	− 2.53	–	44.21	< 0.0001*
SWN–BUSH	− 4.07	–	27.92	< 0.0001*
SWN–CPT	− 5.00	–	9.06	= 0.0026*
SWN–EAV	− 0.89	–	16.24	= 0.0001*
SWN–RBT	− 4.92	–	8.88	= 0.0029*

*Significant difference between methods

^a^Dash (–) in cells indicates missing values of − log likelihood, indicating that a suboptimization step failed to converge. In these cases, a Wald test statistic was used and a *P*-value provided rather than a likelihood ratio test

When collecting male mosquitoes, the SWN and EAV methods performed better than the other methods overall (model contrasts: *χ*^2^ > 6.1, *P* < 0.0134 in all cases). There was some seasonal variation and more males were captured with the SWN method compared to the EAV method at the start of the rainy season (*χ*^2^ = 16.2, *P* < 0.0001), but not at its peak (*P* > 0.05) (Fig. 6).

### Mosquito yield per man/hour effort

**Fig. 7:**
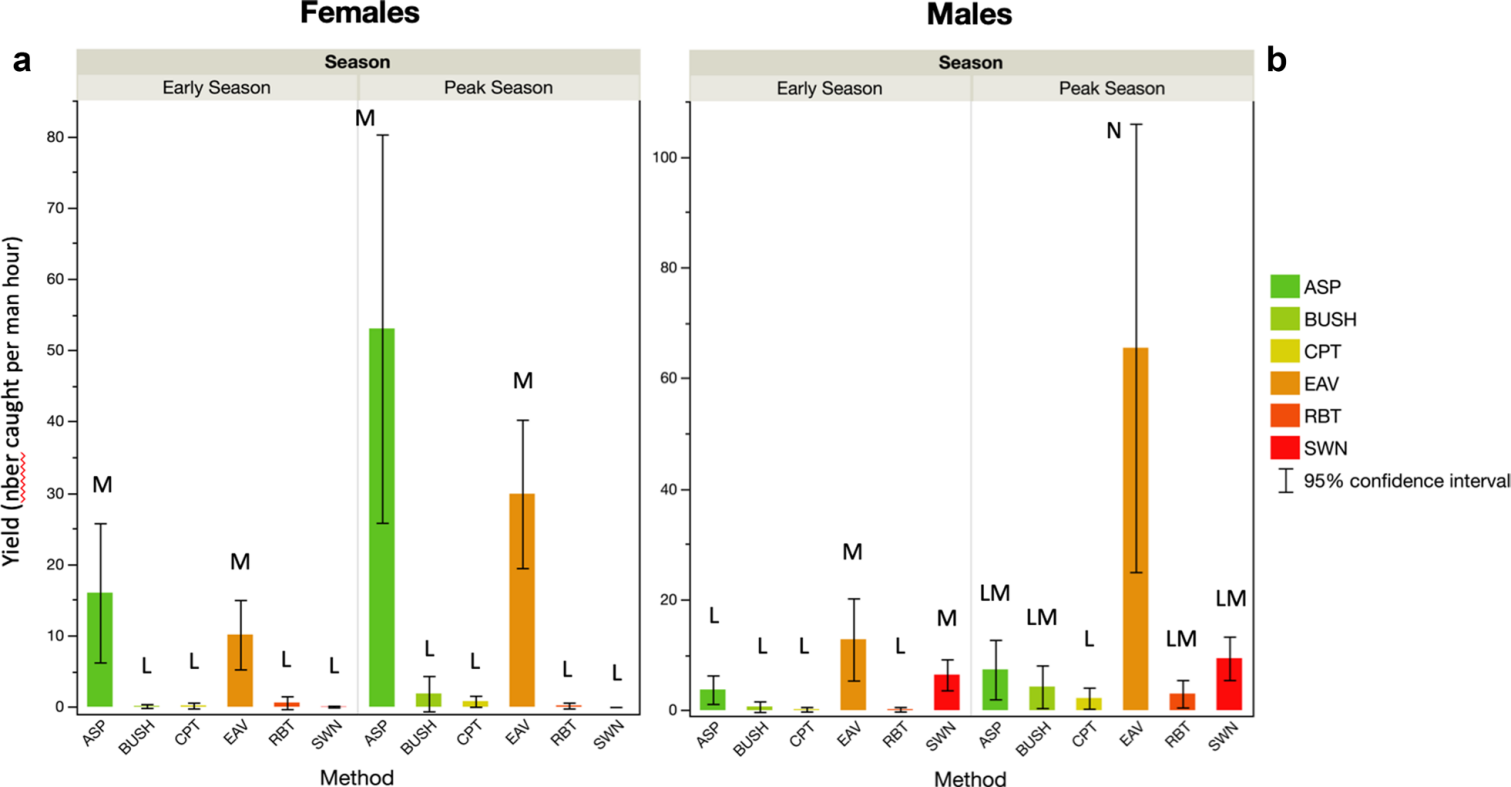
Mean numbers of females (**a**) and males (**b**) calibrated for capture effort (man hours) by season. Bars labelled with different letters were significantly different (model contrast: P < 0.05)

Female mosquito yield (i.e. the number of female mosquitoes caught per unit of collection corrected for man hour effort) did not differ between villages but did vary between seasons and with the method used (Tables 7, 8). Yield per man hour was higher at the peak of the rainy season than at the start (Tables 7, 8; Fig. 7). Overall, the ASP and EAV methods yielded more females per man hour than the other methods tested (model contrasts: *χ*^2^ = 57.9, *P* < 0.0001 for all pairwise comparisons). This pattern was observed both at the start and peak of the rainy season (*P* < 0.001 in all cases) (Fig. 7a), although at the peak of the rainy season, female yield by ASP was higher than that of EAV (*χ*^2^ = 6.5, *P* < 0.0108).

**Table 7:** Summary statistics of the generalized linear model used to estimate the influence of season, village and method on the yield per man hour of *A. gambiae* female and male mosquitoes collected

Sex	Source	*DF*	L–R Chi square^a^	*P*-value
Females	Season	1	37.5	< 0.0001
	Village	2	1.7	0.4362
	Method	5	261.5	< 0.0001
Males	Season	1	50.7	< 0.0001
	Village	2	24.9	< 0.0001
	Method	5	179.2	< 0.0001

Main effects and statistically significant, or near-significant, interactions are shown

^a^Likelihood ratio Chi-squared statistic

**Table 8:** Post-hoc pairwise comparisons (generalized linear model contrasts) of the yield per man hour of *A. gambiae* female and male mosquitoes collected using the different methods

Comparison	Value	Log-likelihood	Chi-square	*P-*value
Females				
BUSH–ASP	3.55	170.57	120.43	< 0.0001*
CPT–ASP	4.24	160.08	99.46	< 0.0001*
CPT–BUSH	0.69	110.58	0.45	= 0.5009
EAV–ASP	0.55	114.90	9.08	= 0.0026
EAV–BUSH	− 2.10	141.44	62.17	< 0.0001*
EAV–CPT	− 3.69	137.35	53.99	< 0.0001*
RBT–ASP	4.46	160.92	101.13	< 0.0001*
RBT–BUSH	0.92	110.71	0.70	= 0.4015
RBT–CPT	0.22	137.35	0.03	= 0.8737
RBT–EAV	3.91	138.06	55.41	< 0.0001*
SWN–ASP	6.86	159.84	98.97	< 0.0001*
SWN–BUSH	3.31	111.68	2.66	= 0.1032
SWN–CPT	2.62	110.86	1.02	= 0.3128
SWN–EAV	6.31	138.93	57.16	< 0.0001*
SWN–RBT	2.39	110.73	0.76	= 0.3844
Males				
BUSH–ASP	0.86	128.38	3.48	= 0.0620
CPT–ASP	1.58	129.99	6.73	= 0.0095
CPT–BUSH	0.72	127.17	0.30	= 0.2989
EAV–ASP	− 1.91	154.86	56.45	< 0.0001*
EAV–BUSH	− 2.77	178.57	103.87	< 0.0001*
EAV–CPT	− 3.49	173.20	93.13	< 0.0001*
RBT–ASP	1.29	129.22	5.18	= 0.0229
RBT–BUSH	0.43	126.86	4.43	= 0.5006
RBT–CPT	0.12	126.69	0.12	= 0.7311
RBT–EAV	3.20	170.99	88.72	< 0.0001*
SWN–ASP	− 0.11	126.67	0.07	= 0.7933
SWN–BUSH	− 0.96	128.85	4.43	= 0.0353
SWN–CPT	− 1.69	130.54	7.80	= 0.0052
SWN–EAV	1.80	150.73	48.20	< 0.0001*
SWN–RBT	− 1.40	129.72	6.16	= 0.0131

*Significant difference between methods

Male mosquito yield was greater at peak season and differed between villages (Tables 7, 8). Once again, the method had the largest effect on the yield per man hour (Tables 7, 8; Fig. 7). For males, SWN and EAV showed the higher collection efficiency at the start of the rainy season, while all the other methods performed similarly (*χ*^2^ < 8.1, *P* < 0.0044) (Fig. 7). However, at the peak of the rainy season, EAV had a higher yield than all other collection methods (*χ*^2^ > 31.0, *P* < 0.0001), achieving the capture of > 60 male mosquitoes per hour of sampling (Fig. 7).

### Effect of house construction type on yield

The house construction type affected the yield of house aspirations for males (Kruskal–Wallis: *χ*^2^ = 25.0, *P* < 0.001). Houses built with mud walls and grass-thatched roofs led to higher yields of male mosquitoes than houses built with brick walls and iron sheet roofs or mud walls and iron sheet roofs (Dunn pairwise comparisons: |*Z*|> 3.5, *P* < 0.0014 in both cases). In contrast, female yield was not affected by the type of house construction (Kruskal–Wallis: *χ*^2^ = 4.0, *P* = 0.1353) (Table 9).

**Table 9:** Efficiency of the house aspiration methods as a function of house type

House type	Interior aspiration (ASP)		Eave aspiration (EAV)		
	Male	Female	Male		Female
Brick/iron sheet	4.6 ± 17.1	37.5 ± 86.7	1.0 ± 2.0	16.0 ± 17.3	
Mud/grass thatch	12.0 ± 14.7	15.5 ± 23.9	46.2 ± 87.9	21.0 ± 24.7	
Mud/iron sheet	4.9 ± 7.9	37.8 ± 48.8	5.3 ± 9.4	14.7 ± 21.3	

Efficiency values are presented as the mean total (± standard deviation) number of mosquitoes per man hour

## Discussion

The mosquito collection methods used in this study proved highly specific to *Anopheles* mosquitoes. Very few other genera were collected during this investigation other than in occasional mixed swarms during the early rainy season.

Aspiration of eaves (EAV) is a method often ignored in population studies. This study shows that this method can not only provide a sex-balanced catch, but one that is efficient in terms of mosquito yield per man hour. The method does require consent from house owners/residents but as it does not intrude into private interior space, it may have ethical and practical benefits. For male catches, eave aspiration works effectively and efficiently in areas of traditional grass-roofed housing, although it may be less useful in the suburban and urban areas where thatched roofs are less frequent. In these latter areas it is possible that resting traps, such as buckets and clay pots, will prove to be more rewarding sampling tools for male catches than in the rural context studied here.

**Fig. 8:**
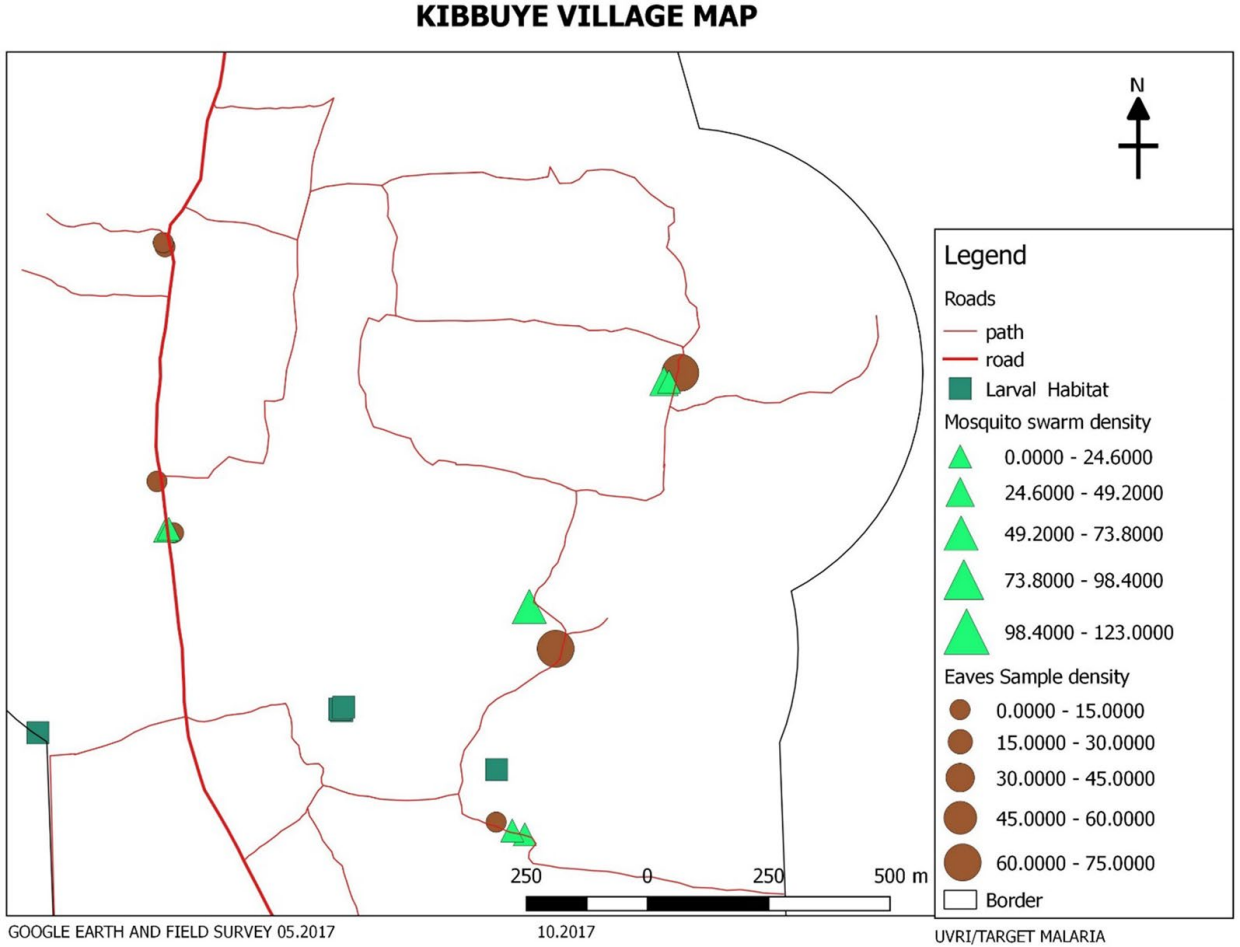
Sample map of Kibbuye village indicating the spatial correlation noted between eave sample and swarm capture density

Unexpectedly, eave aspiration revealed a spatial coincidence between highly productive aspirated house eaves and independently identified mosquito swarm locations (Fig. 8). Using this observation has enabled reliable prediction of the general location of mosquito swarms in subsequent collections. Further study will be needed to understand whether this is a reliable correlation in other areas. If it proves so, rapidly executed eave sampling is likely to be a useful tool for assisting with locating swarms in future collections and may substantially reduce the challenges of finding mosquito swarms in East Africa.

Bush aspiration (BUSH) showed improved performance in terms of numbers of male mosquitoes collected, making up 2.54% (32 mosquitoes) of the total catch at the peak of the rainy season compared to only 0.86% (5 mosquitoes) at the start of the rainy season (*χ*^2^ = 12.17, *P* = 0.033) (Fig. 6). There was a yield per man hour of four mosquitoes during the peak of the rainy season compared to less than one at the start of the season (*χ*^2^ < 3.14, *P* = 0.08) (Fig. 7). Overall bush aspiration remained a disappointing collection method, with very low numbers of male mosquitoes captured. This could be due to the local ecology of these mosquito species where it is possible that they do not prefer to rest in bushes.

Clay pots and buckets used as resting traps have proven successful at capturing both female and male mosquito spp. elsewhere [22, 23], although they were substantially less productive than anticipated during this study. Several factors may have contributed to this: (i) differences in the local *Anopheles* species composition—previous studies captured mostly *A. arabiensis* while the sites in this study were dominated by *A. gambiae* (*s.s.*); (ii) the rural villages had an abundance of bushy vegetation and cool eaves to provide the mosquitoes with a plentiful choice of natural resting places; (iii) both types of resting traps were novel in the environment and there may have been residual and potentially unappealing odors from their manufacturing process. Additionally, the resting traps were not left in the study locations for extended periods. Unlike the other methods tested, neither resting trap method proved effective at the peak of the rainy season, which suggests that male mosquito ecology changes when populations are more abundant and mating opportunities more likely. Perhaps in these situations, male mosquitoes select for different resting shelters, such as house eaves. Further evaluation is required to assess whether the poor performance seen here may be attributed to these factors and whether simple additions such as lures might increase their utility in a rural context.

The male-targeted methods used in this study that were able to capture both sex-balanced and male-dominated samples, such as eave aspiration, and the two types of resting traps could greatly improve our understanding of male mosquito ecology in other contexts. The variation in the numbers of female mosquitoes caught by the methods compared in this study could be explained by the combined effect of low absolute numbers of females, relatively low sample sizes and the fact that these methods are explicitly aimed at males.

In the peak of the rainy season, sweep netting of swarms (SWN) was the most effective method for catching substantial numbers of males, with aspiration of eaves (EAV) coming second. However, in the early season this situation was reversed, and the eaves of grass-roofed houses were particularly rewarding. This highlights the importance of method selection for catching male *A. gambiae *(*s.l.*) mosquitoes and suggests that using several complementary methods may be key to obtaining sufficient numbers for accurate population indices as the seasonal ecology of males varies.

In this study the substantial increase in catch numbers of both males and female mosquitoes at the peak of the rainy season contrasted with the findings of some other studies which found higher population numbers at the start of the rainy season [31–33]. These results suggest that although mosquito populations could be adversely affected by heavy rains, leading to washing away of larvae during peak rainy seasons [32], the formation of additional sites might outweigh the loss of larvae from pre-existing and potentially washed-out sites. It is also possible that there are site-specific rainfall thresholds above which peak-season declines occur. Only time-series data of mosquito numbers and accompanying rainfall data would allow this to be investigated.

All of the male-targeted methods were associated with yields of higher proportions of male *A. gambiae* mosquitoes when compared to the frequently used indoor aspiration methods. Indoor targeted methods catch many females that are resting after taking blood meals or which are attracted by host odors. This result further affirms that specific male-targeted methods are required to boost male mosquito capture proportions in traditional mosquito population sampling. Of the methods evaluated, swarm collection and aspiration of house eaves offered particular value to sampling designs aimed to give a comprehensive description of the mosquito population dynamics, and demonstrably delivered a higher male proportion than interior aspirations (ASP). These were also the most effective in terms of numbers of individuals captured per unit, indicating that effective sampling for studies and surveillance of anophelines can be predictably achieved through aspiration sampling of a limited number of randomly chosen house eaves or swarms. In contrast, although aspiration of bushes (BUSH) and resting traps (RBTs, CPTs) yielded sex-balanced samples, the results could also require consideration of specific ecological circumstances. The low return per sample would translate into very large numbers of bushes and resting traps being needed to obtain adequate numbers. This would then be logistically cumbersome.

These differences in trapping efficiency and practicality are further highlighted when human resources are taken into account. Many factors contribute to the overall ‘effort’ of sampling mosquitoes in the field: planning, logistics, stakeholder engagement, staff time, volunteer training, consumables, among others. The metric in this study did not encompass all of these factors and provided only an estimation of the mosquito yield of these methods as a specific function of the field-man hours. Nonetheless, this value was useful and indicated greater efficiency of male capture at the peak of the rainy season when compared to lower background densities earlier in the season. The same was not true for females, possibly indicating in general a higher efficiency of the methods used for collecting females, especially the female-targeted interior aspiration (ASP) method. However, this is only one possible explanation, and further study would more clearly evaluate the relative efficiency of capture method between sexes and seasons. It was interesting to note that although sweep netting of swarms did catch high numbers, aspiration of grass-roofed houses collected in the villages gave the highest returns of male *A. gambiae* mosquitoes for the least effort. The substantial effort involved in locating and sampling swarms reduced its efficiency relative to other methods. This emphasizes that being clear about requirements is crucial to the planning of sampling. When high numbers are needed, then the effort of swarm sampling may be necessary; if a representative index of males and females is sought, then aspiration of bushes and eaves may serve well.

It can be deduced that the sampling times may be flexible. The paucity of mosquitoes found in afternoon aspiration samples (1 mosquito found in 223 samples) and other methods (EAV, RBT, CPT and BUSH) used to sample a location for a second time on the same day affirm that the mosquitoes did not relocate during the late morning hours. This lack of daytime movement suggests that it may be possible to relax the time at which sampling is performed, or to extend the hours of sampling if conditions permit. Further optimization and experimentation may be required in order to fully understand whether the time of collection does influence the productivity or effectiveness of these methods. While these observations may be of use for those planning sampling, they did not contribute any evidence to the discussion surrounding behavioral shifts of anopheline mosquitoes towards activity earlier in the evening.

The house type of selected sampling units may have been more influential on the catch obtained. The male mosquito catch was greater from the grass thatch-roofed houses than from the tin sheeted-roofed ones, but as this was not observed for the female catch it may indicate that different features are attractive to each sex. Females may be drawn by the prospect of blood meals regardless of roof type, whereas males, looking for a resting site for daytime shelter, may find the cooler, and likely less variable, shade of thatched roots more attractive than that of tin roofs. This would affect the selection of an efficient sampling design where house type is variable—as can be found in many of the rural villages in Uganda.

## Conclusions

*Anopheles gambiae* mosquito swarms could be located and used to collect males in Uganda. The mosquito swarm collection method was the most productive male mosquito collection method tested in this study, while house eave aspiration was the most efficient. The clear efficiency of house eave aspiration in delivering a sex-balanced and man–hour efficient catch is extremely useful to the design of mosquito collection experiments. This is particularly true for population studies, such as MRR experiments, and in study designs where sex-balanced results are important. This study provided the useful observation that aspiration of eaves could prove to be a better indicator of *A. gambiae* mosquito swarming locations in East Africa than the identification of physical markers, which is a technique largely informed by West African studies. There are many locations where *A. gambiae* mosquito swarms remain elusive and further investigation is warranted. Male collection methods are clearly a research direction with plenty of room left for further exploration, innovation, improvement and optimization of available methods. With the progression of new and innovative technologies, such as the sterile insect technique, and active genetics studies which rely on a greater understanding of male mosquito dynamics and behavior, this work is increasingly important and timely.

## Data Availability

The data are part of a wider study of baseline mosquito abundance across Africa and are available from the corresponding author on reasonable request.

## Abbreviations

ASP: Aspiration of house interiors
BUSH: Aspiration of bushes
CPT: Clay pot trap
EAV: Aspiration of house exterior eaves
RBT: Resting bucket trap
SWN: Sweep net collection of swarms
